# Five-Year Clinical Outcomes of Surgically Treated Different Types of Tibial Pilon Fractures

**DOI:** 10.2106/JBJS.OA.26.00277

**Published:** 2026-10-27

**Authors:** Timo Lenkkeri, Ville Bister, Mika Koivikko, Jan Lindahl

**Affiliations:** 1 Department of Orthopaedics and Traumatology, Helsinki University Hospital and University of Helsinki, Helsinki, Finland; 2 Department of Surgery, Clinicum, Faculty of Medicine, University of Helsinki, Helsinki, Finland; 3 Department of Radiology, HUS Diagnostic Center, Helsinki University Hospital and University of Helsinki, Helsinki, Finland

## Abstract

**Background:**

Intra-articular distal tibial pilon fractures are most commonly caused by high-energy trauma and are often associated with severe soft-tissue injuries. The literature on medium-to-long-term clinical outcomes is limited. The purpose of this follow-up study was to evaluate the 5-year clinical outcomes and patient satisfaction among patients with different types of tibial pilon fractures.

**Methods:**

One hundred twenty-six consecutive patients with operatively treated tibial pilon fractures were identified between 2012 and 2016 from a level 1 trauma center. A total of 105 patients (mean age 43 years, 30 females, 100% White, 100% European) were eligible for follow-up and were contacted 5 years after the initial trauma. Outcomes were assessed using a questionnaire, Foot and Ankle Outcome Score (FAOS), American Orthopaedic Foot and Ankle Society (AOFAS) score, functional tests, and posttraumatic osteoarthritis (OA) Kellgren–Lawrence (K–L) grade. For outcome analysis, patients were categorized into the following 3 groups according to AO-OTA fracture classification: type 43B2-B3 (Group 1), type 43C1-C2 (Group 2), and type 43C3 (Group 3) tibial pilon fractures.

**Results:**

No statistically significant differences between study groups were observed in FAOS (mean 74, standard deviation [SD] 16.9) and AOFAS (mean 82, SD 12.7) scores. Posttraumatic OA (K–L grade 3-4) was diagnosed in 35% in Group 1, 8% in Group 2, and 48% in Group 3. Ten patients (8%) underwent ankle arthrodesis. The incidence of deep surgical-site infections increased with severity of fracture type (no patients in Group 1, 21% in Group 2, and 23% in Group 3). Two patients (1.6%) required below-the-knee amputation. Approximately 90% of the healthcare resources were needed within the first 2 years after injury; patients in Group 3 required the most surgeries and had the longest hospitalization.

**Conclusions:**

Surgically treated pilon fractures seem to result in good mid-term clinical outcomes, even for severe C3 fractures. However, type C3 fractures had more complications and required more surgical interventions, including ankle arthrodeses, longer hospitalization, and additional other healthcare resources than the less severe fractures.

**Level of Evidence:**

Therapeutic Level III. See Instructions for Authors for a complete description of levels of evidence.

## Introduction

Intra-articular distal tibial fractures comprise <1% of all lower extremity fractures and 3% to 10% of tibial fractures^1-6^. These fractures are commonly caused by high-energy trauma, and patients often have concomitant injuries^1-3^. Severe soft-tissue injuries are often involved^7-9^, and multiple surgical procedures are frequently required^8-11^. Temporary external fixation and delayed internal fixation with locking plates or definitive treatment with ring fixator are the preferred techniques for managing high-energy pilon fractures^12^. In complicated cases, early soft-tissue reconstruction may be needed^8,11^.

There are limited reports on medium-to-long-term results^13-16^. In some studies, patients with e.g., severe soft tissue injuries, open fractures, polytrauma, or comorbidities have been excluded from the study population. Tibial pilon fractures are associated with devastating, long-term effects on patient-reported outcomes, and health-related quality of life^12,14,17,18^. High rates of infection complications and posttraumatic osteoarthritis (OA) have also been reported^5,9,13,19-22^.

The purpose of this study was to evaluate the medium-term clinical outcomes of different types of tibial pilon fractures. In addition, we sought to evaluate the burden of these injuries to the individual patient and to the public healthcare system up to 5 years after the initial trauma. Our hypothesis was that the AO-OTA type C3 fractures have the worst outcomes and need more healthcare resources compared with the type B2-B3 and C1-C2 fractures.

## Materials and Methods

Permission for this study was provided by the Ethical Board of Helsinki University Hospital (HUS/2188/2017).

### Patients

One hundred twenty-six consecutive patients with operatively treated intra-articular distal tibial fracture (AO-OTA 43 B2-B3 and C1-C3) were retrospectively identified between 2012 and 2016 from a level 1 trauma center. Most (92%) patients were treated with locking-plate osteosynthesis aiming for the best possible articular surface reduction and reestablished alignment, and 89% of the patients were treated with a staged (controlled delay) treatment protocol^23^. Patients were divided into the following 3 groups according to AO-OTA 43 fracture classification^24^: Group 1, type B2-B3; Group 2, type C1-C2; and Group 3, type C3 tibial pilon fractures.

### Study Design and Follow-Up Protocol

Patients were seen at follow-up visits conducted 5 years after the trauma. At the follow-up visit, an interview on symptoms and a clinical examination were performed by the physiotherapist. Weight-bearing ankle radiographs were taken, and patient-reported outcome measures (PROMs) were collected.

Two-year follow-up results of the same study population have been published previously, including patient demographics, injury mechanisms, and initial treatment (Supplementary Table 1)^23^. Race and ethnicity data were extracted from the institution’s database. Patients who had not undergone ankle arthrodesis or below-the-knee amputation were invited for a 5-year follow-up visit. The study flowchart is presented in Fig. 1.

**Fig. 1:**
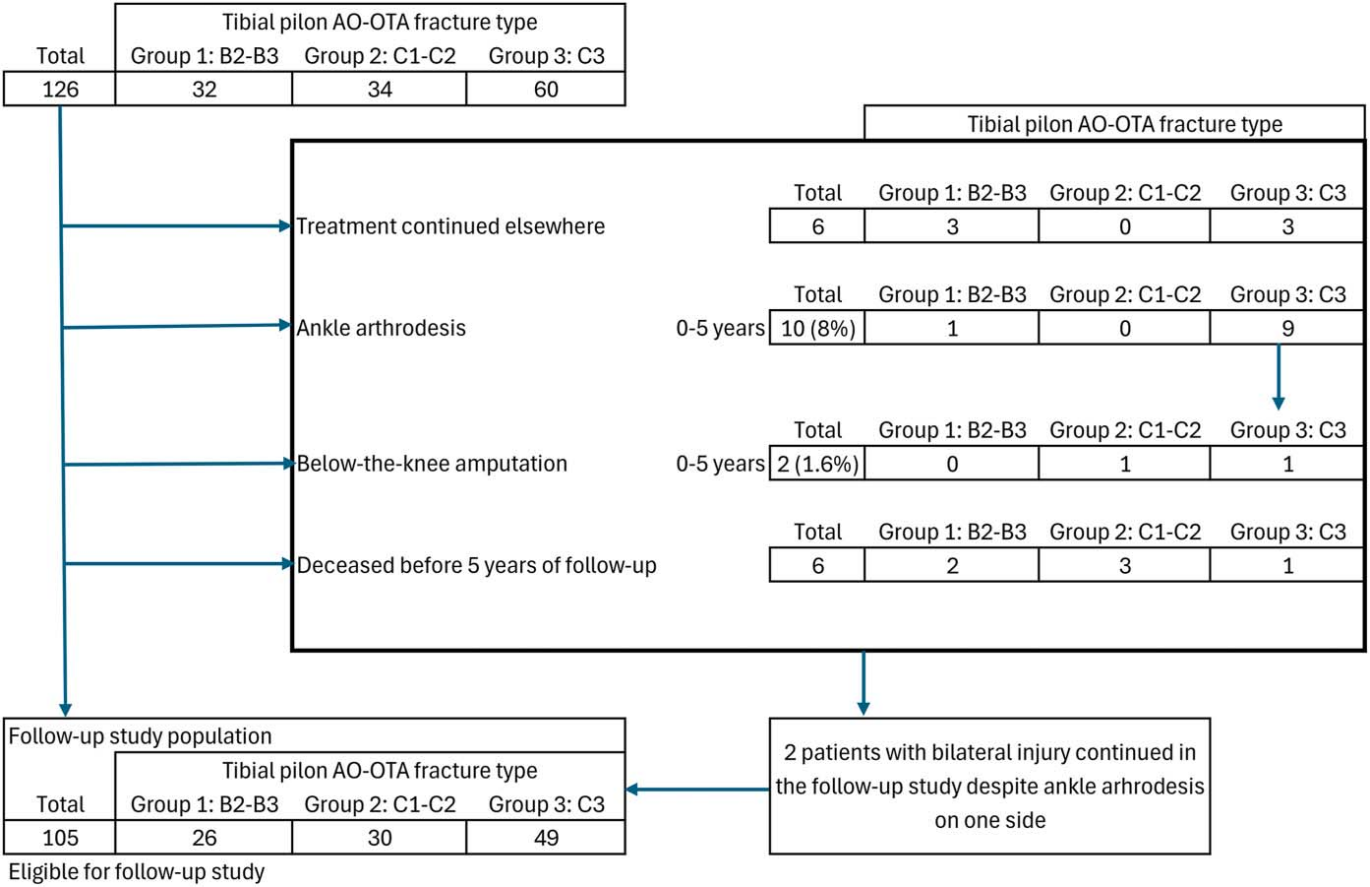
Study flowchart.

### Outcomes

Subjective satisfaction was evaluated with Foot and Ankle Outcome Score (FAOS) and American Orthopaedic Foot and Ankle Society (AOFAS) Ankle-Hindfoot Score. Posttraumatic OA Kellgren-Lawrence (K–L) grade was diagnosed with weight-bearing ankle radiographs. Ankle range of motion (ROM) and toe raise tests were performed. Working status was inquired.

### Adverse Events/Complications

Data on adverse surgical complications (deep surgical-site infection [SSI], nonunion, deep venous thrombosis, neurological complications, and below-the-knee amputation) and minor complications (superficial SSI) were collected. SSIs were classified as deep if all of the following criteria existed simultaneously: clinical signs of infection, positive bacterial cultures from the wound or blood, and fixation material palpable or visible in the wound^25^. K–L grade 3 to 4 posttraumatic OA was defined as significant^26^.

### Healthcare Resources Needed

Data on surgeries, in-hospital-days, outpatient clinic visits, and lengths of treatment periods were collected.

### Statistical Analysis

Statistical analysis was performed using Microsoft Excel and IBM SPSS Statistics v28. An independent-samples Kruskal-Wallis test was used for comparison of means for continuous variables, which were nonnormally distributed (FAOS, AOFAS). Statistical significance was set at p < 0.05.

## Results

### Patient-Reported and Clinical Outcomes

No statistically significant differences between Groups 1, 2, and 3 were observed at 5-year postinjury in FAOS score, FAOS subgroup scores, or AOFAS results (Table 1). Clinical examinations did not show clear differences in functional test results between groups (Table 2).

**Table 1:** FAOS and AOFAS Scores in Different Tibial Pilon Fracture Group 5-Year Postinjury (Mean)

	All	Tibial Pilon AO-OTA Fracture Type			p
		Group 1: B2-B3	Group 2: C1-C2	Group 3: C3	
FAOS total (0-100)*	74	72	78	73	0.538
Pain	80	77	82	82	0.615
Symptoms	62	55	70	61	0.114
Activities and daily living	84	83	87	83	0.831
Sport	57	60	62	52	0.279
Quality of Life	61	62	65	58	0.536

	All	Group 1: B2-B3	Group 2: C1-C2	Group 3: C3	p
AOFAS total (0-100)†	82	80	87	81	0.210
Pain (0-40)	30	29	31	30	0.670
Function (0-50)	44	42	46	43	0.073
Alignment (0-10)	9	9	9	8	0.570

AOFAS = American Orthopaedic Foot and Ankle Society, and FAOS = Foot and Ankle Outcome Score.

* FAOS at 5-year follow-up (n = 82).

† AOFAS at 5-year follow-up (n = 69).

**Table 2:** Ankle Range of Motion and Toe Raises in Different Tibial Pilon Fracture Group 5-Year Postinjury

		Tibial pilon AO-OTA fracture type		
	All	Group 1: B2-B3	Group 2: C1-C2	Group 3: C3
Average active ROM in the ankle (degrees)*	43	41	50	39
Average loss of ankle ROM compared with the healthy ankle (degrees)†	16	16	11	19
Average toe raises on the injured foot (number of raises)‡	21	21	24	20
Average deficit in toe raises compared with the healthy ankle (number of raises)§	5	6	2	8

ROM = range of sagittal motion.

* n = 69.

† n = 65, bilateral injuries excluded.

‡ n = 69, toe raises on the injured foot to the beat of a metronome (1 raise per second).

§ n = 65, bilateral injuries excluded.

### Fracture Healing

Reliable data on time to union were available for 102 patients (23 in Group 1, 30 in Group 2, and 49 in Group 3). Bony union and consolidation of fracture lines and gaps were observed in 52 (51%) patients within 6 months and in 78 (76%) patients within 1 year of injury. Fracture healing took longer than 1 year in 24 (24%) patients. Bony union took longer in Group 3 than in Groups 1 and 2 (Fig. 2).

**Fig. 2:**
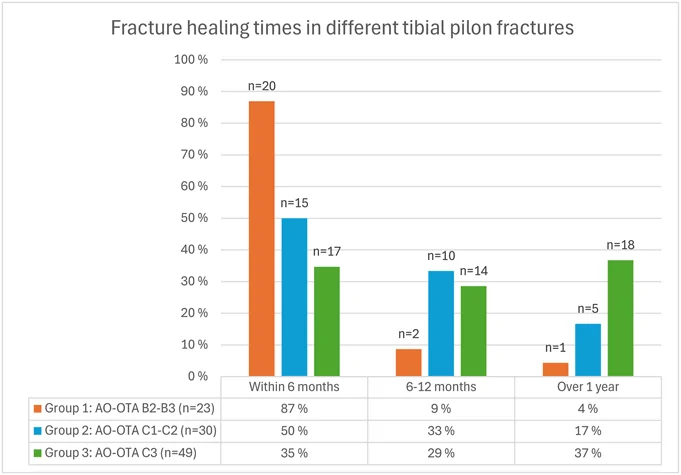
Time to union in radiographs in different tibial pilon fracture groups (n = 102).

### Posttraumatic Osteoarthritis

Posttraumatic ankle OA K–L grade 3 to 4 was observed in 29 of 88 (33%) patients. The risk of getting posttraumatic OA within 5 years after injury was 48% (19/40) in Group 3, 8% (2/25) in Group 2, and 35% (8/23) in Group 1. Ten (8%) patients underwent ankle arthrodesis (Fig. 1). Nine of the arthrodeses (90%) were performed in Group 3, which was 15% of all Group 3 patients.

### Return to Previous Work

Eighty-two patients reported their work status at the 5-year follow-up visit. Twenty (24%) patients had changed profession after the injury (24% [5/21] in Group 1, 24% [6/25] in Group 2, and 25% [9/36] in Group 3). No clear differences in back-to-work rates were seen between groups.

### Adverse Effects/Complications

Owing to nonunion, surgical treatment was required for 17 of 126 (13%) patients. Of these patients, 12 (71%) also had infection-related issues. Thirteen of 17 (76%) patients needing surgery due to nonunion were in Group 3. The nonunion rate in Group 3 was 22% (13/60). Four (12%) patients in Group 2 underwent surgery due to nonunion; no patients in Group 1 underwent surgery due to nonunion.

The incidence of deep SSI was highest in Group 3 (14/60, 23%). Table 3 presents the observed complications in the 5 years after trauma in different tibial pilon fracture types. Two patients required a below-the-knee amputation (1 in Group 3 and 1 in Group 2). Both amputation patients had posttraumatic chronic osteomyelitis.

**Table 3:** Complications within 5 Years from the Injury Related to Different Types of Tibial Pilon Fractures (n = 126)

	All Patients (%)	Tibial Pilon AO-OTA Fracture Type		
		Group 1: B2-3	Group 2: C1-2	Group 3: C3
Superficial wound infections*	16 (13)	6 (19%)	2 (6%)	8 (13%)
Deep surgical-site infections*	21 (17)	0	7 (21%)	14 (23%)
Nerve injuries*	15 (12)	4 (13%)	3 (9%)	8 (13%)
Thromboembolism-related complications*	4 (3)	0	0	4 (7%)

* % of patients in the study group.

### Healthcare Resources Needed

A total of 126 patients required 369 surgical procedures over the 5-year follow-up period. Most surgeries (339/369, 92%) were performed within 2-year postinjury. Mean numbers of surgeries required were 1.9 (Group 1), 2.2 (Group 2), and 3.9 (Group 3). Sixty-four (51%) patients required additional surgery after definitive osteosynthesis. Twenty (16%) patients required soft-tissue reconstruction. Of these, 17 (85%) were in Group 3. In Group 3, 17 of 60 (28%) patients needed soft-tissue reconstructions. Implant removal was performed on 31 of 126 (25%) patients.

Of the outpatient clinic visits, 87% (758/868) occurred within 2 years after injury. Mean visits required were 4.6 (Group 1), 6.1 (Group 2), and 8.5 (Group 3). Most (92/110, 84%) of the visits after 2 years were required by patients in Group 3. Mean length of treatment period was 14.5 months (Group 1, 7.3 months; Group 2, 13.0 months; and Group 3, 19.2 months). Mean number of days in hospital was 21 (Group 1, 14; Group 2, 17; and Group 3, 27).

## Discussion

There were no significant differences in patient-related outcome measures, subjective satisfaction, return-to-work rates, or functional measurements between different types of tibial pilon fractures at 5 years after injury. These results were observed even though patients in Group 3 had more infection complications, nonunion, posttraumatic OA, and required approximately twice the amount of healthcare resources compared with the other study groups. Approximately 90% of the healthcare resources were needed within the first 2 years after injury. Moreover, a substantial proportion of the healthcare resources needed 2 years after injury were required for treatment of patients in Group 3.

Previous studies have reported mean FAOS scores of 76 and mean AOFAS scores of 78 to 87 at mid-term follow-up after open reduction and internal fixation of tibial pilon fractures^13,27^. Our results are consistent with these previous studies. Different conditions and injuries are not fully comparable using PROM instruments. Outcomes of tibial pilon fractures can nevertheless be contrasted with reference values and with outcomes reported for other injuries. Our FAOS results are worse than FAOS reference values in a national representative sample in Denmark^28^, meaning that tibial pilon fractures have an impact on these patients’ life. A study of operatively treated ankle fracture dislocations with a mean follow-up of 41 months showed about the same FAOS results as we found in our material for tibial pilon fractures at 5 years^29^.

The rate of deep SSI varies from 2% to 24% in pilon fracture patients^20-22,30-34^, which is consistent with our results (17%). In our material, most of the deep SSIs were detected in Groups 2 and 3 patients, which can be explained by high-energy trauma and more severe soft-tissue injuries. Several risk factors for surgical site infections in patients with pilon fractures have been identified^21^. Our results are consistent with these findings regarding the open fracture status and the AO-OTA fracture type.

In our study, return-to-previous work rates were better than in previous reports^14,35^. In our material, 24% of patients did not return to their previous work. Pollak et al. reported that 43% remained unemployed at a mean of 3.2 years after injury^14^.

Group 3 patients needed the most healthcare resources and had approximately twice as many surgeries than the other groups, with 28% requiring soft-tissue reconstruction procedures, of which 85% were in Group 3. Our results emphasize the need for good orthoplastic cooperation and soft-tissue reconstructions in complex injuries with infection complications, which has also been recommended previously^11,23^.

The risk for posttraumatic OA appeared to be low in Group 2, where the fracture in the articular surface is simple. The risk for posttraumatic OA within 5 years after injury increased up to 48% in Group 3. In our study, 33% had radiologically significant posttraumatic OA (K-L 3-4), and approximately one-third of these required ankle arthrodesis. In the entire material, 8% needed ankle arthrodesis and 90% of these were in Group 3. This appears to be consistent with the previously observed 15% in a mean follow-up of 10 years^15^.

We regarded the need for ankle arthrodesis as an undesirable outcome. Previous studies have reported FAOS scores of 57 to 64 at mid-term follow-up after ankle arthrodesis for posttraumatic OA^36,37^. In our study, the FAOS results of patients treated with osteosynthesis were better than those reported for ankle arthrodesis performed for posttraumatic OA.

There was no primary ankle arthrodesis in this study. This might be an option in some rare high-energy AO-OTA type C3 tibial pilon fractures with severe comminution of the articular surface. The indications for primary ankle arthrodesis in tibial pilon fractures are unclear. The indications have included severe articular cartilage injury or severely comminuted fractures that have been considered non-reconstructable usually by the treating surgeon^5,12,38,39^. A systematic review concluded that primary ankle arthrodesis yields reasonable outcomes in pilon fracture management, but larger studies with longer follow-up are warranted^40^.

Satisfactory functional outcomes are achievable in appropriate patients using ORIF even in the most severe tibial pilon fracture types. In some individual patients, severe injury might lead to a poor outcome. It is important to inform patients that treatment is demanding and time-consuming.

This study has some inherent limitations concerning the patient chart analysis. There may be some underreporting, as some complications, adverse events, and resource use may have been undetected. Furthermore, patients with arthrodesis, which are probably the most severe cases, were excluded from the prospective follow-up study. This may affect the findings concerning the functional outcomes of tibial pilon fractures in general. In our study, we did not observe differences in functional outcomes between the study groups among those patients who had managed with osteosynthesis for 5 years. It is possible that with a larger sample size, a statistical or clinically significant difference might have emerged. Owing to the rarity of the injury, however, obtaining a sufficiently large cohort to achieve adequate power is challenging.

The strengths of this study include the relatively large study population of tibial pilon fractures (including consecutive patients), a long follow-up period of 5 years, patient-reported outcome measurements and clinical examinations planned at 5 years after injury, and comprehensive reporting of resources used. To our knowledge, this is one of the largest cohort studies with at least 5 years of follow-up.

The results of the current study support the treatment of high-energy tibial pilon fractures with a staged surgical protocol. The most complicated fractures require good cooperation with orthopaedic trauma surgeons and plastic surgeons from the start. Future research should focus especially on the AO-OTA type C3 pilon fractures and their treatment methods, as the challenges and resource utilization are particularly pronounced in this group.

In conclusion, the most severe AO-OTA type C3 fractures had more infection complications, nonunion, posttraumatic OA, and needed more healthcare resources than the less severe type B2-B3 and type C1-C2 fractures. Nevertheless, surgical management of tibial pilon fractures seems to result in relatively good patient-related and clinical outcomes without significant differences between fracture subtypes.

## Appendix

Supporting material provided by the authors is posted with the online version of this article as a data supplement at jbjs.org (https://links.lww.com/JBJSOA/B381). This content was not copyedited or verified by JBJS.
